# The Bul1/2 Alpha-Arrestins Promote Ubiquitylation and Endocytosis of the Can1 Permease upon Cycloheximide-Induced TORC1-Hyperactivation

**DOI:** 10.3390/ijms221910208

**Published:** 2021-09-22

**Authors:** Amalia H. Megarioti, Cecilia Primo, George C. Kapetanakis, Alexandros Athanasopoulos, Vicky Sophianopoulou, Bruno André, Christos Gournas

**Affiliations:** 1 Microbial Molecular Genetics Laboratory, Institute of Biosciences and Applications, National Centre for Scientific Research “Demokritos”, Patr. Grigoriou E & 27 Neapoleos St., 15341 Agia Paraskevi, Greece; amaliam@bio.demokritos.gr (A.H.M.); alexandr@bio.demokritos.gr (A.A.); vicky@bio.demokritos.gr (V.S.); 2 Molecular Physiology of the Cell Laboratory, Université Libre de Bruxelles (ULB), IBMM, 6041 Gosselies, Belgium; Cecilia.Primo.Planta@ulb.be (C.P.); Georgios.Kapetanakis@ulb.be (G.C.K.)

**Keywords:** ubiquitin, α-arrestin, endocytosis, transporter, arginine, Target of Rapamycin Complex 1, Npr1, acidic patch, Nedd4

## Abstract

Selective endocytosis followed by degradation is a major mechanism for downregulating plasma membrane transporters in response to specific environmental cues. In *Saccharomyces cerevisiae,* this endocytosis is promoted by ubiquitylation catalyzed by the Rsp5 ubiquitin-ligase, targeted to transporters via adaptors of the alpha-arrestin family. However, the molecular mechanisms of this targeting and their control according to conditions remain incompletely understood. In this work, we dissect the molecular mechanisms eliciting the endocytosis of Can1, the arginine permease, in response to cycloheximide-induced TORC1 hyperactivation. We show that cycloheximide promotes Rsp5-dependent Can1 ubiquitylation and endocytosis in a manner dependent on the Bul1/2 alpha-arrestins. Also crucial for this downregulation is a short acidic patch sequence in the N-terminus of Can1 likely acting as a binding site for Bul1/2. The previously reported inhibition by cycloheximide of transporter recycling, from the trans-Golgi network to the plasma membrane, seems to additionally contribute to efficient Can1 downregulation. Our results also indicate that, contrary to the previously described substrate-transport elicited Can1 endocytosis mediated by the Art1 alpha-arrestin, Bul1/2-mediated Can1 ubiquitylation occurs independently of the conformation of the transporter. This study provides further insights into how distinct alpha-arrestins control the ubiquitin-dependent downregulation of a specific amino acid transporter under different conditions.

## 1. Introduction

In all cells, transporters (or permeases) are essential components of the plasma membrane. Most of them catalyze the selective uptake of nutrients, including carbon and nitrogen sources, ions, and vitamins. Work in bacteria from the 1960s has importantly contributed to current knowledge about how these proteins function. Ronald Kaback was a pioneer and a leading figure in the field. The exhaustive work combining physiology, genetics, and biochemistry that he carried out with his collaborators has long popularized LacY, the *Escherichia coli* lactose permease, as the most-thoughtfully characterized transporter [1]. Transporters are very often subject to tight control, as initially illustrated by regulation of LacY cell-surface expression via transcriptional control of the *lac* operon [2]. Subsequent studies in eukaryotes showed that plasma membrane transporters may additionally be post-translationally regulated, in particular at the level of their intracellular trafficking. Important contributions to this field were provided by studies of several fungal transporters [3]. Inhibition of these proteins in response to environmental changes most often occurs through endocytosis followed by sorting to the vacuole where they are degraded [4]. This downregulation is initiated by the covalent addition of ubiquitin (Ub) to specific Lys residues of the transporter [5]. In the yeast *Saccharomyces cerevisiae*, this ubiquitylation is generally mediated by the Rsp5 Ub-ligase [6], the sole member of the Nedd4 family of Ub-ligases in this species. Rsp5 preferentially catalyzes linkage of K63-linked poly-Ub chains [7]. This type of ubiquitylation was shown to be particularly important for sorting internalized cargo proteins to multi vesicular bodies (MVBs) via the ESCRT machinery, a crucial step for their subsequent delivery to the vacuolar lumen and degradation [5,8,9]. Recruitment of Rsp5 to target transporters is mediated by the arrestin-like (or alpha-arrestin or ART family) adaptor proteins [4,10,11,12,13,14]. The genome of *S. cerevisiae* codes for 14 alpha-arrestins, some of which correspond to paralogs. Accumulating evidence supports that the specificity of Rsp5 towards its target membrane proteins is dictated by these alpha-arrestins. Specifically, the ability of these proteins to interact with their targets or Rsp5 itself is thought to be regulated by their ubiquitylation [15,16,17,18,19] and phosphorylation status [16,20,21,22]. The latter has also been suggested to control the topology of the ARTs [20]. Phosphorylation of several alpha-arrestins was shown to occur in response to specific nutritional or stress conditions and via pathways involving specific signaling complexes, such as TORC1, Snf1/AMPK, calcineurin, and TORC2 [15,16,20,23,24,25,26,27]. It is also well established that PPXY motifs in alpha-arrestins are recognized by the WW-domains of Rsp5 [10,14,15,16,28]. However, how the alpha-arrestins recognize the transporter cargos has been investigated in only a few cases [28,29,30,31,32,33,34]. Notable is the presence of “acidic patch” sequences in the cytosolic N- and/or C-termini of transporters, suggested to act as recognition sites by several alpha-arrestins [30,32]. Moreover, alpha-arrestins not only act at the plasma membrane, but also at the trans-Golgi network (TGN) or even endosomes [20,21,22,29]. Furthermore, evidence suggests that de-ubiquitylation of transporters by specific enzymes might promote their recycling back to the plasma membrane via the TGN [19,35,36,37,38,39].

**Figure 1:**
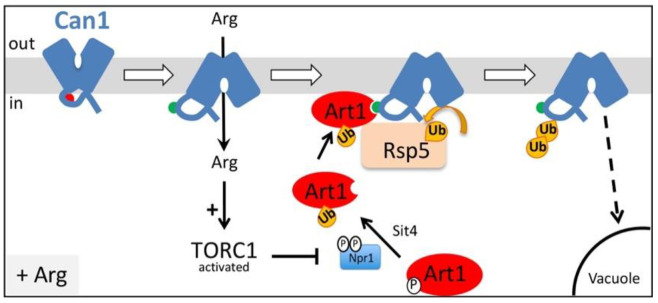
Model for Art1-dependent Can1 endocytosis in response to Arg transport. In the absence of substrate, Can1 is mostly found in an outward-facing conformation and this structures the N-terminal tail in a way that masks the binding site for Art1 (aa 70–81, red hemicycle). Once Arg is added in the medium, a transient shift of the permease to an inward-facing conformation of the transport cycle repositions the N-terminal tail and exposes the binding site for Art1 (now green hemicycle). Arg uptake also stimulates TORC1, leading to inactivation of Npr1, and activation of Art1 via de-phosphorylation. Activated Art1 can then recognize the exposed binding site at the N-tail of Can1, resulting in Rsp5-mediated ubiquitylation of Can1, endocytosis and sorting to the vacuole for degradation.

The Arg permease Can1 is one of the eukaryotic transporters whose downregulation by endocytosis has been best studied. This process was reported to occur under diverse conditions, including the presence of its substrate Arg [17,29,40], addition of cycloheximide [20], several stress conditions [41,42], and starvation for carbon (glucose) [26,43] or nitrogen [32]. We have recently dissected at the molecular level the mechanism inducing ubiquitylation and endocytosis of Can1 in response to Arg transport. Our results demonstrated that primarily Art1 and secondarily the Bul1/2 alpha-arrestins act as adaptors of Rsp5 for Can1 ubiquitylation induced by the presence of substrate (Figure 1) [17,29]. More specifically, efficient Can1 downregulation upon Arg transport is initiated by Art1-dependent ubiquitylation, for which two conditions need to be fulfilled [29]: 1) a shift of Can1 to an inward-facing conformation as occurs during transport catalysis, causing exposure to the cytosol of a short N-terminal Art1-binding sequence (Art1-BS) and 2) activation of Art1 via the amino acid-responsive TORC1 cascade [20]. Another study reported similar conclusions regarding the substrate-induced endocytosis of the methionine permease Mup1 [30]. Importantly, the authors of this study have also provided genetic evidence for the existence of an Art1-BS at the N-terminus of Mup1 that interacts with a sequence in Art1 enriched in basic amino-acids. This Art1-BS sequence of Mup1 is mentioned as an “acidic patch” due to the prominent presence of acidic amino acid residues. Similar “acidic patch” sequences required for transporter endocytosis are found at the N-tails of several fungal transporters [44], including Fur4 [45], Gap1 [17], Tat2, and Lyp1 [32], but also at the C-tails of the uric acid transporter UapA [28], the lactate transporter Jen1 [31], and the amino acid transporters Mup1, Can1, Tat2, and Lyp1 [32]. Acidic patches at the C-tails of Mup1, Can1, Tat2, and Lyp1 have been shown to be required for the endocytosis of these permeases in response to nitrogen starvation, and most likely correspond to binding sites for the Art2 α-arrestin [32].

Our previous study of Arg transport-elicited Can1 ubiquitylation also unraveled a minor role of the Bu11 and Bul2 adaptors. We have obtained evidence that these proteins act through a Can1 N-terminal sequence likely corresponding to their binding site (Bul-BS) [29]. This Bul-BS seems constitutively accessible to the Bul1/2 adaptors, since activation of TORC1 by addition of ammonium (Am) was sufficient to provoke Bul1/2-dependent Can1 ubiquitylation (an Arg-induced conformational change of the transporter was not required in this case). However, contrary to Arg-induced ubiquitylation mediated by Art1, this Arg-induced Bul1/2-dependent ubiquitylation does not lead to efficient sorting of the permease to the vacuole for degradation, and Can1 instead recycles back to the plasma membrane via the trans-Golgi Network (TGN). Thus, the physiological significance of Bul1/2-mediated ubiquitylation of Can1 remains elusive. We have also previously shown that the Bul1/2 adaptors are regulated by the TORC1-Npr1 signaling pathway in response to nitrogen availability, and subsequently control the ubiquitylation and turnover of the general amino acid permease, Gap1 [16,17]. More specifically, activated TORC1 phospho-inhibits the Npr1 kinase, which inhibits by phosphorylation the Art1 and Bul1/2 alpha-arrestins [16,20]. Hence, upon TORC1 activation, Art1 and Bul1/2 get activated by relief of Npr1-mediated phospho-inhibition. Other recent works reported a role of Bul1 in the endocytosis of Jen1, the lactate transporter, in response to glucose [46], alkalization of the external medium, or addition of cycloheximide (CHX) [47]. CHX causes hyperactivation of TORC1 [48,49], and thus of Art1 via inhibition of Npr1 [20], and this is probably due to the over-accumulation of free amino acids provoked by blockage of protein synthesis [48,50]. Importantly, Art1 has been previously reported to participate in the CHX-induced endocytosis of Can1 [20], since the permease is partially protected from endocytosis in an *art1*Δ strain [10]. This seems, at first, inconsistent with more recent models supporting that TORC1 activation alone is not sufficient to promote the recognition of Can1 or Mup1 by Art1, as a shift in the conformation of the transporter is additionally required in order to unmask the Art1-BS [29,30].

In this work, we further investigate the role of the Bul1/2 in ubiquitylation and endocytosis of Can1. We show that, in the absence of Can1 substrates in the medium, CHX-induced ubiquitylation, endocytosis, and vacuolar sorting of Can1 are mediated solely by the TORC1-activated Bul1/2 adaptors acting through the Bul-BS at the N-terminus of the transporter. Moreover, we propose that a defect in transporter recycling could additionally contribute to CHX-induced vacuolar sorting of Can1. Finally, we provide further evidence that Can1 downregulation via Bul1/2 does not require a shift of the transporter to an inward-facing conformation, which contrasts with when it is mediated by Art1.

## 2. Results

### 2.1. The Bul α-Arrestins Promote Cycloheximide-Induced Endocytosis of Can1

**Figure 2:**
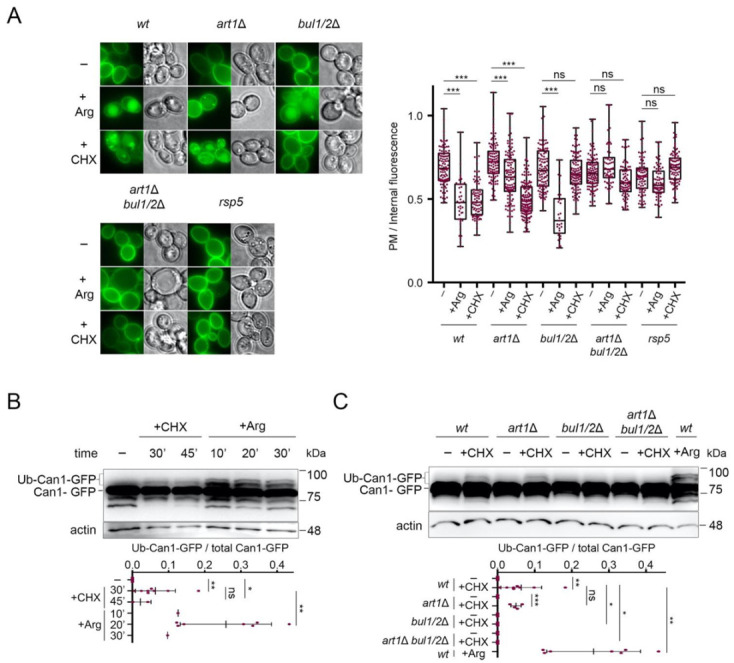
Can1 endocytosis in CHX-treated cells requires the Bul1/2 alpha-arrestins. (**A**) Epifluorescence microscopy of Can1-GFP in wt, *bul1/2*Δ, *art1*Δ, *art1*Δ *bul1/2Δ*, and *npi1-1* strains. Strains were grown on Minimal Medium (MM) with Galactose (Gal) and Ammonium (Am). GAL1-promoter-driven expression of CAN1-GFP was repressed by addition of 3% Glucose (Glu) for 1 h. Arg (5 mM) or CHX (50 μg/mL) were then added for 3 h. Quantifications: plasma membrane (PM) to internal GFP fluorescence intensity ratios, are plotted (*n* = 31–135 cells) as box and whiskers plots. The horizontal midlines represent the median values, the boxes are bounded by the upper and lower quartiles, and the whiskers denote the maximal and minimal ratios. Red dots represent the individual values. ***, *p* < 0.001; ns, nonsignificant, *p* > 0.05. Scale bar is 2 μm. (**B**) Wt strain expressing Can1-GFP was grown on Gal, Am MM. Glu was added for 30 min, a non-treated sample was collected (-) and then CHX was added for 30 or 45 min or Arg for 10-, 20- or 30-min. Total protein extracts were probed with antibodies against GFP and actin. The low mobility bands correspond to Ub-Can1-GFP conjugates. Quantifications: The ratio of Ub-Can1-GFP / Total Can1-GFP are plotted in scattered plots. Red dots represent the individual biological replicates. ***, *p* < 0.001; **, 0.001 < *p* < 0.01; * 0.01 < *p* < 0.05; ns, nonsignificant, *p* > 0.05. Lower exposure is provided in supporting information. (**C**) The indicated strains expressing Can1-GFP were grown on Gal, Am MM. Glu was added for 30 min, a non-treated sample was collected (-) and then CHX was added for 30 min or Arg for 20 min. Protein extracts were probed and analysed as in B. Lower exposure is provided in supporting information.

In order to study the effect of cycloheximide (CHX) on Can1 molecules solely present at the plasma membrane and not at internal compartments, we expressed wt and mutant *CAN1* alleles from the galactose-inducible and glucose-repressible *GAL1* promoter. Cells were thus initially grown on galactose as the sole carbon source, and prior to CHX or Arg addition, *CAN1* expression was repressed by the addition of glucose for sufficient time to allow all synthesized Can1-GFP molecules to reach the plasma membrane [17]. The nitrogen source used was Ammonium, the preferred nitrogen source, leading to full activation of nitrogen catabolite repression in the Σ1278b strain [51,52], contrary to the situation in S288C and its derivatives [53,54]. Under this experimental setup, pre-synthesized Can1-GFP was indeed exclusively detected at the plasma membrane (Figure 2A). Subsequent addition of CHX or Arg promoted a reduction of the fluorescent signal at the plasma membrane and concomitant appearance of fluorescence at internal bright spots (presumably endosomes and TGN [22,29,55]) and at the lumen of the vacuole (Figure 2A). In line with our previous findings [29], Arg-induced Can1 endocytosis was unaltered in a strain lacking the Bul1/2 adaptors, severely impaired in a strain lacking Art1, and totally blocked only in a strain lacking all three Art1, Bul1, and Bul2 adaptors (Figure 2A). On the contrary, while CHX induced similar vacuolar sorting of Can1 in the wt and *art1*Δ mutant strains, it failed to do so in all strains lacking the Bul1/2, whether these expressed Art1 or not. The same protection from CHX-induced internalization was observed in a npi1-1 hypomorph mutant of Rsp5, previously shown to be defective for Can1 and Gap1 ubiquitylation [6,17] (Figure 2A).

In support of the view that Can1 undergoes CHX-induced ubiquitylation, we were able to detect by Western blotting slow-migrating Can1-GFP bands shortly after CHX treatment (Figure 2B). These upper bands, although less intense and thus more difficult to detect, displayed the same electrophoretic mobility as those typically observed upon Arg addition (Figure 2B) and previously shown by immunoprecipitation to correspond to Ub-Can1-GFP conjugates [29]. Furthermore, they were not detectable in protein extracts from CHX-treated strains lacking the Bul1/2 adaptors (Figure 2C). Thus, our results indicate that CHX promotes Can1 endocytosis via Rsp5-mediated ubiquitylation, and that this modification involves recognition of the permease by the Bu1/2 alpha-arrestins.

### 2.2. Cycloheximide-Induced Endocytosis of Can1 Requires an Intact Putative Bul1/2 Binding Sequence and the Ub-Acceptor Lys^42^ and Lys^45^ in the Permease N-Tail

**Figure 3:**
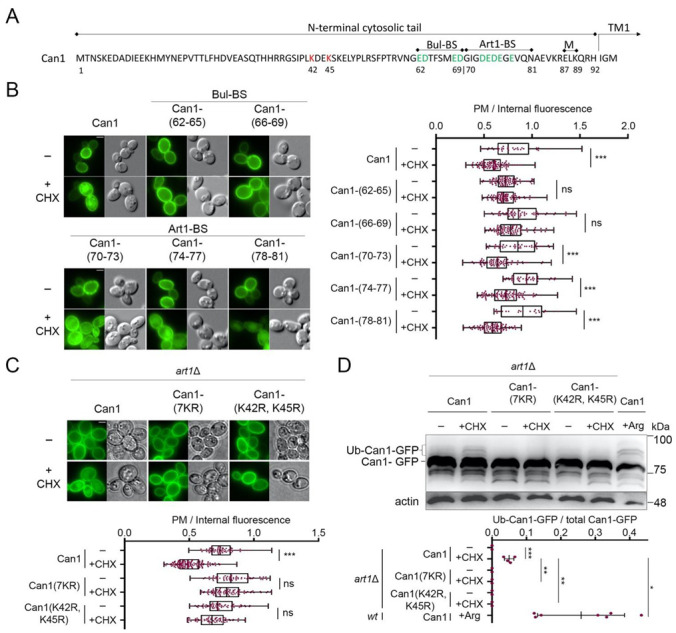
Cycloheximide-induced endocytosis of Can1 requires Lys^42^ and Lys^45^ residues and an intact putative Bul-BS at the permease N-tail. (**A**) Amino acid sequence of the N-terminus of Can1. The main Ub-acceptor Lys^42^ and Lys^45^ are highlighted. Residues 62–69 and 70–81 have been identified as the putative binding sites of the Bul1/2 and Art1 α-arrestins, respectively. These sequences are enriched in negatively charged amino acids (highlighted in green). Residues 87–89, previously shown to be important for masking the Art1-BS in the outward-facing conformation [29], are also highlighted. (**B**) Epifluorescence microscopy of strains expressing *wt* Can1-GFP or Can1-GFP mutants carrying Ala-substitutions at the indicated residues of the N-tail. These residues of Can1 comprise the probable binding sites for Art1 (Art1-BS) or Bul1/2 (Bul-BS). Strains were grown on Gal, Am MM, Glu was added for 60 min and then CHX for 3 h before imaging, as in Figure 2A. Quantifications: Plasma membrane (PM) to internal GFP fluorescence intensity ratios are plotted (*n* = 25–101 cells). Quantifications, representations and scale bar as in Figure 2A. (**C**) Epifluorescence microscopy of *art1*Δ cells expressing Can1-GFP or the indicated Lys-to-Arg substitution alleles. Glu was added for 90 min and then CHX for 3 h before imaging, as in Figure 2A. Quantifications: Plasma membrane (PM) to internal GFP fluorescence intensity ratios are plotted (*n* = 70–135 cells). Quantifications, representations and scale bar as in Figure 2A. (**D**) *art1*Δ cells expressing the indicated Can1-GFP alleles were grown on Gal, Am MM. Glu was added for 30 min, a non-treated sample was collected (-) and then CHX or Arg was added for 30 or 20 min, respectively. Total protein extracts were probed and analysed as in Figure 2B. Lower exposure is provided in supporting information. The low mobility bands correspond to Ub-Can1-GFP conjugates. Quantifications: The ratio of Ub-Can1-GFP/Total Can1-GFP are plotted in scattered plots. Red dots represent the individual biological replicates. ***, *p* < 0.001; **, 0.001 < *p* < 0.01; * 0.01 < *p* < 0.05; ns, nonsignificant, *p* > 0.05.

We then focused on identifying *cis* elements required for the endocytosis of Can1 by CHX. For this, we adopted an unbiased approach similar to the one that previously allowed us to identify sequences in the N-terminus of Can1 required for Arg-induced endocytosis of the permease via Art1 or Bul1/2 [29]. More specifically, we employed an Alanine-scanning mutagenesis of the N-tail of Can1 (Figure 3A). Twenty-three Can1 mutants have previously been constructed, for each of which 3–4 consecutive residues have been substituted by Alanine (Ala) residues. These Can1 mutants have been introduced in a *can1*Δ strain and examined for CHX-induced endocytosis. Using this approach, we observed that most mutants, including those altered in the predicted Art1-BS (residues 70–81), underwent normal endocytosis (Figure 3B and Figure S1). Three others, however, were found to be resistant to CHX-induced internalization. One of them carries Ala substitutions of residues 42–45, a region in the N-tail (Figure 3A), which was further investigated (see below). The two others carry Ala substitutions of residues 62–65 and 66–69, respectively (Figure 3A). Importantly, the N-terminal region between residues 62–69 (Figure 3A) corresponds to the one we have previously identified as a putative Bul-BS, as it was required for the Bul-dependent residual Arg-induced ubiquitylation of Can1 detected in the *art1*Δ strain [29]. Thus herein, by applying a different screen, we re-identify residues 62–69 to be essential for Bul1/2-mediated endocytosis of Can1. These results provide further support to the view that the 62–69 residue region comprises the binding site for the Bul1/2 adaptors, and strongly suggest that this putative Bul-BS is involved in both the Arg- and CHX-induced ubiquitylation of Can1. Interestingly, we also noticed that the mutant carrying Ala-substitutions of residues 82–86 shows CHX-induced internalization but significantly reduced vacuolar sorting (Supplementary Figure S1). These residues include one Lys and are located between the Art1-BS and the 87–89 region required for masking the Art1-BS (Figure 3A). They could thus potentially be involved in the Ub-dependent delivery of Can1 from the Golgi to the vacuole, but more work is needed in order to clarify this.

The N-tail region between residues 42 and 45 includes Lys^42^ and Lys^45^, the main redundant Ub-acceptor Lys residues required for Can1 degradation by Arg [29]. In order to verify that Lys^42^ and Lys^45^ are indeed involved in the CHX-induced endocytosis of Can1, we examined by microscopy and Western blotting the effects of CHX on two additional Can1 mutants (Figure 3C,D). The first, Can1(K42R, K45R), carries Lys to Arg substitutions of residues 42 and 45, while the second, Can1(7KR), carries Arg substitutions of all seven Lys residues of the N-terminus of Can1. In both mutants, the addition of CHX did not provoke any detectable increase in Can1 ubiquitylation (Figure 3D). Moreover, the quantification of Can1 signal intensity from microscopy images showed that both mutants were highly resistant to endocytosis. These results indicate that CHX-induced endocytosis of Can1 is mediated solely by ubiquitylation at Lys^42^ and/or Lys^45^. This contrasts with Arg transport-induced endocytosis of Can1, for which additional Lys-residues of the N-tail are most likely ubiquitylated, albeit with lower efficiency [29].

### 2.3. Cycloheximide Promotes Endocytosis of Can1 via TORC1 Hyperactivation and/or Blockage of Transporter Recycling

**Figure 4:**
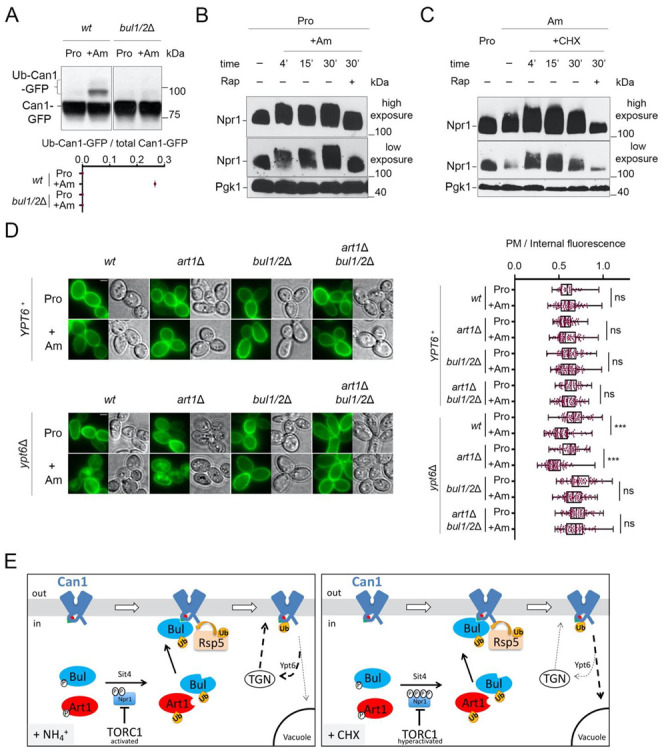
Cycloheximide promotes endocytosis of Can1 via TORC1 hyperactivation and/or blockage of transporter recycling. (**A**) Immunoblots of total protein extracts of *wt* and *bul1/2*Δ cells expressing Can1-GFP. Cells were grown on Gal Proline (Pro) medium. Glu was added for 30 min followed by Am for another 30 min. Total protein extracts were probed and analysed as in Figure 2B. The low mobility bands correspond to Ub-Can1-GFP conjugates. Quantifications: The ratio of Ub-Can1-GFP / Total Can1-GFP are plotted in scattered plots from one biological replicate. (**B**) Immunoblots of total protein extracts of a wild-type strain expressing HA-Npr1 and grown in Pro medium, followed by the addition of Ammonium (Am). Samples with or without rapamycin addition 15 min after Am addition were collected at the indicated time-points and probed with anti-HA and anti-Pgk1 antibodies. High and low exposures are shown. (**C**) Immunoblots of total protein extracts of a wild-type strain expressing HA-Npr1. Cells were grown on Gal Proline (Pro) or Ammonium (Am) medium, followed by the addition of CHX. Samples with or without rapamycin addition 15 min after CHX addition were collected at the indicated timepoints and probed with anti-HA and anti-Pgk1 antibodies. High and low exposures are shown. (**D**) Epifluorescence microscopy of Can1-GFP in *wt*, *bul1*/*2*Δ, *art1*Δ, and *art1*Δ *bul1/2*Δ cells in *YPT6*^+^ or *ypt6*Δ background, grown in Gal Pro medium. Glu was added for 1 h followed by addition of Am for 3 h. Quantifications: Plasma membrane (PM) to intracellular GFP fluorescence intensity ratios are plotted (*n* = 45–101 cells). Quantifications, representations and scale-bars as in Figure 2A. (**E**) Model for the mechanisms controlling the endocytosis of Can1 upon TORC1 activation. Upon addition of Am (Left) in cells grown on a poor nitrogen source, TORC1 gets activated and leads to phosphorylation of Npr1. The resulting inactivation of Npr1 leads to activation of Art1 and the Bul1/2. However, only the Bul1/2 can recognize the Bul binding sequence (residues 62–69, green triangle) since the Art1-BS remains masked (red hemicycle). Can1 ubiquitylation via Rsp5 leads to endocytosis, but subsequent vacuolar sorting is limited because the permease recycles back to the plasma membrane via the Trans-Golgi Network (TGN), in a Ypt6-dependent way. When CHX is added (Right), on the contrary, TORC1 gets hyperactivated, leading to hyper-phosphorylation of Npr1 and activation of Bul1/2 and Art1. At the same time, recycling of Can1 is somehow inhibited, thus the ubiquitylated Can1 is sorted to the vacuole.

The results presented above strongly support the view that ubiquitylation of Can1 in CHX-treated cells is mediated by Rsp5 and involves recognition by Bul1/2 of a specific sequence in the N-tail of Can1. CHX was previously proposed to provoke Can1 endocytosis through activating TORC1 [20]. Consistently, the Bul1/2 adaptors gain the ability to promote permease ubiquitylation when TORC1 is activated by addition of Ammonium (Am), the preferred nitrogen source in the Σ1278b strain [16]. This was also illustrated in the case of Can1 ubiquitylation induced by Am addition to cells grown on L-Proline (Pro), a poor nitrogen source ([29], Figure 4A). However, in contrast to the situation of CHX addition to Am-grown cells, Can1 ubiquitylation in Pro-grown cells supplied with Am does not promote efficient vacuolar sorting of Can1 ([29], see also below). One potential difference between CHX addition to Am-grown cells and Am addition to Pro-grown cells is that TORC1 might be more highly activated in the former cells. Indeed, we obtained evidence in support of this hypothesis by monitoring the phosphorylation of Npr1, a widely used readout of TORC1 activity and a direct mediator of Bul1/2 activity [16,20,56,57]. As previously studied in detail [16,56], the addition of Am to Pro grown cells leads to increased phosphorylation of Npr1, as detected by a typical decrease in the electrophoretic mobility of Npr1 in SDS-PAGE (Figure 4B). This phosphorylation is TORC1-activity-dependent, as it can be impeded by rapamycin, a specific inhibitor of TORC1 [58]. However, while Npr1 phosphorylation was consistently higher when cells grew on Am instead of Pro as a nitrogen source, this phosphorylation was further enhanced by addition of CHX, unless cells were treated with rapamycin (Figure 4C). These results, in keeping with previous data [20], suggest that CHX activates TORC1 to levels which tend to be higher than those caused by Am. This hyperactivation could explain, at least in part, why Can1 downregulation is more pronounced in CHX-treated cells than in Am-supplied cells.

Another potential reason accounting for the more efficient delivery of ubiquitylated Can1 to the vacuole after CHX vs. Am addition is that recycling of membrane cargos back to the plasma membrane could be inhibited by CHX treatment, as reported in a previous study for the Fur4 transporter [38]. Other works reported that internalized transporters can recycle back to the plasma membrane [21,22,29]. One could thus consider that, upon TORC1 activation elicited by Am addition, the Can1 permease, because it undergoes only limited ubiquitylation, would not be efficiently sorted to the vacuole after its internalization, thereby tending to recycle to the plasma membrane. In CHX-treated cells, however, limited ubiquitylation might lead to the more efficient delivery of Can1 to the vacuole because general recycling to the cell surface is hindered. To assess this model, we monitored Am-induced endocytosis of presynthesized Can1-GFP molecules localized at the plasma membrane of mutants carrying the deletion of *YPT6*, which encodes a key factor Rab-GTPase involved in the recycling from endosomes to the TGN [59]. Importantly, in the *ypt6*Δ mutant cells, addition of Am leads to more efficient internalization and vacuolar sorting of Can1. Furthermore, this downregulation proved to be dependent on Bul1/2 but not on Art1 (Figure 4D). We thus tried to take advantage of this condition, to directly compare the rate of CHX- vs Am-induced endocytosis of Can1 in Pro-grown *ypt6*Δ cells. However, we realized that this experiment is not feasible, since CHX addition to Pro-grown cells leads to Can1 endocytosis that is independent of Art1 and the Bul1/2 (Supplementary Figure S2). More work is needed in order to dissect this phenotype.

Overall, our results support the following model (Figure 4E): When TORC1 is stimulated by Am addition in Pro grown cells, the activated Bul1/2 promote the ubiquitylation and endocytosis of Can1. However, this does not lead to efficient vacuolar sorting, most probably because ubiquitylation is too limited or de-ubiquitylation is particularly efficient, causing recycling of Can1 via the TGN. On the contrary, CHX addition in Am grown cells causes a more pronounced activation of TORC1, and at the same time could potentially block transporter recycling [38], eventually leading to more efficient vacuolar sorting of Can1. 

### 2.4. Can1 in the Outward-Facing Conformation Is Not Recognized by TORC1-Activated Art1

**Figure 5:**
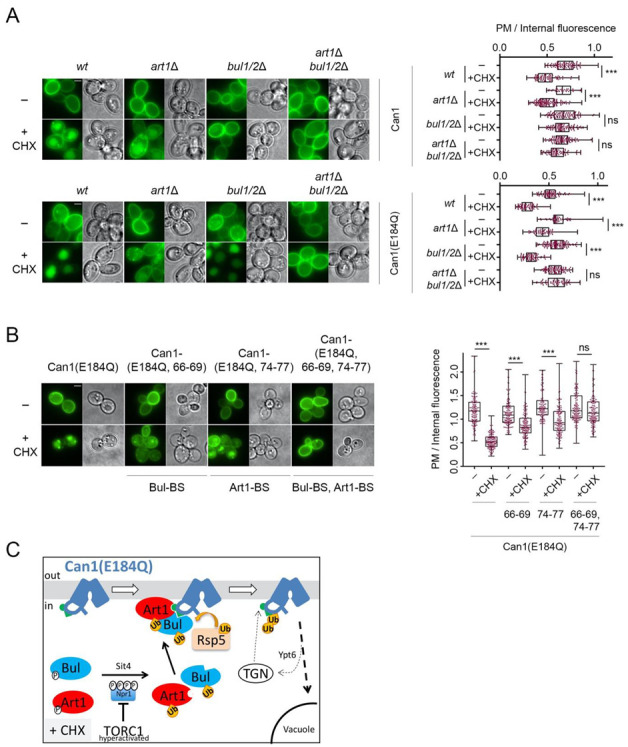
Artificial exposure of the masked Art1-BS at the N-tail of Can1 renders the permease sensitive to TORC1-activated Art1. (**A**) Epifluorescence microscopy of wild type Can1-GFP and Can1(E184Q) -GFP in *wt*, *bul1*/*2*Δ, *art1*Δ, and *art1*Δ *bul1/2*Δ strains. Cells were grown in Gal and Am MM. Glu was added for 1 h followed by addition of CHX for 3 h. Quantifications: Plasma membrane (PM) to internal GFP fluorescence intensity ratios are plotted (*n* = 38–135 cells). Quantifications, representations and scale bar as in Figure 2A. The quantifications for *wt* Can1 are the same from Figure 2A, since the experiments have been performed in parallel. (**B**) Epifluorescence microscopy of *gap1*Δ *can1*Δ cells expressing the indicated Can1 alleles combining E184Q with Ala substitutions in either the Art1-BS (residues 74–77), or the Bul-BS (residues 66–69) or both. Conditions, quantifications (*n* = 128–160) and representations as in A. (**C**) Schematic representation of the results concerning the CHX-induced endocytosis of Can1(E184Q). This allele stabilizes Can1 in an Inward-Facing conformation and constitutively exposes the binding site for Art1 (residues 70–81, green hemicycle). Upon CHX addition, Can1 is recognized not only by the Bul1/2 (Figure 4E), but also by Art1, leading to more efficient ubiquitylation by Rsp5, endocytosis and degradation.

A previous study reported that Art1, the primary alpha-arrestin involved in the substrate-induced ubiquitylation and endocytosis of Can1 [17,29], is required for CHX-induced Can1 endocytosis [20]. In contrast, the results presented herein show that in our conditions, Art1 is not involved in the CHX-induced endocytosis of Can1. These results are also in line with our previous findings that Art1 promotes efficient Can1 ubiquitylation and down-regulation only when the permease shifts to an inward-facing (IF) conformation, which exposes the Art1-BS at the N-tail (Figure 1, [29]). Hence, in the absence of Arg and the presence of CHX, TORC1-hyperactivated Art1 cannot in principle recognize Can1 because the Art1-BS remains masked (Figure 4E). A prediction compatible with this model is that TORC1 activation by CHX treatment should promote efficient Art1-dependent ubiquitylation and endocytosis of a Can1 protein whose Art1-BS is exposed to the cytosol (and thus to activated Art1) even in the absence of Arg. To assess this prediction, we took advantage of a particular inactive Can1 allele, Can1(E184Q). We have previously provided evidence—using structural modeling, ubiquitylation/endocytosis assays, and confocal microscopy-based microdomain partitioning [29,60]—that Can1(E184Q) is stabilized in an inward-facing conformation and thereby constantly exposes the Art1-BS. The results presented in Figure 5A clearly show that Can1(E184Q) is hypersensitive to CHX-induced endocytosis. Most importantly, this hypersensitivity of Can1(E184Q) to CHX is due to Art1 and not the Bul1/2 adaptors, since it is not reduced in *bul1/2*Δ cells, strongly indicating that Art1 is able and very efficient to promote the vacuolar delivery of this particular Can1 mutant. Consistently, the hypersensitivity of Can1(E184Q) to CHX is reduced in *art1*Δ cells to levels comparable with the Bul1/2-dependent vacuolar sorting of *wt* Can1 but is strongly impaired in the triple *art1*Δ *bul1/2*Δ strain. Results in the same direction have been obtained by combining in *cis* the E184Q substitution with Ala-substitutions in the Art1-BS and/or the Bul-BS (Figure 5B). Total protection from CHX-induced endocytosis was only evident for the mutant combining the E184Q with mutations in both the Art1-BS and Bul-BS. In keeping with these observations, a Can1 mutant bearing Ala-substitutions in residues 87–89, previously shown to be required for masking the Art1-BS (Figure 3A) [29], is also hypersensitive to CHX (Supplementary Figure S1) and this depends on Art1 (data not shown). The above results in total strongly indicate that Art1 is activated upon CHX addition and able to mediate Can1 ubiquitylation only if the Art1-BS is exposed (Figure 5C).

## 3. Discussion

In this work, we uncover the molecular mechanisms driving the endocytosis of the Arg permease Can1 by CHX. Consistent with previous reports [20], we show that CHX causes a hyperactivation of TORC1, reflected by a strong increase of Npr1 phosphorylation (Figure 4C). TORC1-mediated phosphorylation of Npr1 is well known to inactivate the kinase, while a main role of Npr1 is to phospho-inhibit alpha-arrestins such as Bul1/2 and Art1 [16,20,56]. Thus, the observed hyperphosphorylation of Npr1 by TORC1 is expected to lead to a hyper-activation of Βul1/2 and Art1 [16,20], known to be involved in Can1 downregulation [10,17,29]. However, our results support that when TORC1 is activated under conditions where Can1 substrates are unavailable in the external medium, only Bul1/2 are able to promote Can1 endocytosis, and they do so via a small putative Bul-BS sequence (residues 62–69) at its N-terminal tail (Figure 4E). This endocytosis is induced by Rsp5-mediated covalent attachment of Ub at residues Lys^42^ and/or Lys^45^ of Can1 and results in efficient vacuolar sorting of Can1 for degradation. The mechanism is thus very similar to the downregulation of the general amino acid permease Gap1 in response to Ammonium-induced TORC1 activation [16].

Our results showing that TORC1-stimulated Art1 does not contribute to Can1 endocytosis upon CHX treatment contradict those reported previously [10,20]. This apparent discrepancy can be reconciled by taking into account that Art1 participates in the substrate transport-induced endocytosis of Can1 (Figure 1) [10,17,29]. In this case, however, the trigger of permease ubiquitylation is not only activation of Art1 by TORC1, but also a shift of Can1 from an outward-facing to an inward-facing conformation, unveiling the Art1-BS. This conformational switch occurring during Arg transport could also happen when Lys or His, which are lower affinity substrates of Can1 [61] and common supplements in yeast growth media for complementing auxotrophies, are present in the external medium. Under these conditions, Can1 molecules in an inward-facing conformation are likely more populated and thus more prone to ubiquitylation and downregulation via Art1 upon TORC1 activation by CHX treatment. This view is fully supported by our results showing that Art1 promotes particularly efficient CHX-induced endocytosis of the inactive Can1(E184Q) mutant that is stabilized in an inward-facing state, thereby constantly exposing the Art1-BS (Figure 5C). The presence of Can1 substrates in the medium could potentially favor Can1 ubiquitylation and endocytosis via another mechanism, namely exit of the protein from specialized domains of the plasma membrane called the Membrane Compartment of Can1 (MCC) [62]. Within this domain, Can1 and other transporters have been found to be protected from ubiquitylation and endocytosis, while they tend to diffuse out of them upon substrate transport [26,60,63,64,65,66,67,68], which should thus make them more accessible to the TORC1-activated Art1. 

Our results provide further clarification concerning how distinct amino acid transporters respond differently to favorable nitrogen supply conditions. Specific transporters have been shown to localize at the plasma membrane only when cells are grown on poor nitrogen sources (i.e., when TORC1 is lowly active), and to be efficiently endocytosed when TORC1 is stimulated upon addition of a good nitrogen source like Ammonium. These include Put4, the transporter of the poor nitrogen source Pro [69,70], and the general amino acid permease Gap1, a transporter of broad specificity acting as amino acid scavenger [71]. On the contrary, there is another category of transporters displaying high specificity, namely Can1, Mup1, Tat2, and Lyp1, which are known to be at the plasma membrane even in the presence of ammonium, i.e., conditions of high TORC1 activity [17,29,32,61,72,73]. These transporters are endocytosed when two conditions are met: presence of their substrate, and stimulation of Art1 via activated TORC1 [17,29,30,32]. This mechanism has been proposed to add specificity to the endocytosis of these transporters, ensuring that only those whose substrate is available are endocytosed, in order to avoid toxicity due to the over-accumulation of a certain amino acid [29]. The results presented herein suggest that the difference in the sensitivity of a given transporter to TORC1-stimulation is determined not only via recognition by different alpha-arrestins, but also by differential sensitivity to the same alpha-arrestins. More specifically, it seems that Gap1 is highly sensitive to Bul1/2, and gets endocytosed with high efficiency even when these alpha-arrestins are activated by Ammonium [16,17,74]. On the contrary, Can1—and potentially also Mup1, Tat2, and Lyp1—seems to be less sensitive than Gap1 to Bul1/2, and a hyperactivation of TORC1 is in this case required in order to achieve efficient downregulation. How this differential sensitivity to the Bul1/2 is determined remains to be clarified, and this could be related to the nature of the Bul-BS at the N-termini of the two transporters, or to the efficiency of de-ubiquitylation and recycling (see also below). Nevertheless, the differential regulation of these transporters likely reflects the different roles they accomplish in the cell. The amino acid scavenging role of Gap1 in nitrogen-starved cells is no-longer advantageous under nitrogen-replete conditions. On the contrary, accumulation at the plasma membrane of transporters for specific amino acids even in nitrogen-rich conditions can be beneficial under certain conditions. Indeed, the supply of certain amino acids is known to be beneficial for growth during fermentation. For example, Arg supplementation in *Schizosaccharomyces pombe* can reduce the dependence of amino acid biosynthesis on mitochondrial activity [75], while Met and Lys uptake allows for the reduction of NADPH consumption coupled to the biogenesis of both amino acids, resulting in a beneficial antioxidant effect [76]. Interestingly, Can1, Mup1, and Lyp1, the permeases specific for these amino acids, have all been shown to partition to the MCC [40]. Partitioning of Can1 and Mup1 to MCCs is known to protect the permeases from ubiquitylation and endocytosis [60,63], while at least for Can1 this protection is known to occur at the stationary phase or upon glucose starvation, ensuring optimal growth recovery upon nutrient re-supply [26,60].

Finally, it would be interesting to understand in detail the metabolic effects caused by CHX addition and leading to TORC1 hyperactivation. The blockage of protein synthesis by CHX has been previously suggested to lead to high-level accumulation of free internal amino acids [48,50]. Cells could naturally encounter such a situation when amino acids are highly concentrated in the extracellular medium. This condition has been shown to be potentially toxic for yeast cells [77]. Thus, massive endocytosis of transporters in response to TORC1 hyperactivation might have been naturally selected to avoid amino acid toxicity. In addition to TORC1 hyperactivation, CHX treatment could potentially block the recycling of Can1 back to the plasma membrane (Figure 4), similarly to previously published results about Fur4 [38]. The underlying mechanism, however, is not known and future work will be needed to characterize whether this is a more general phenomenon. A potential explanation could be that the neosynthesis of a high-turnover protein needs to be maintained for supporting efficient recycling of membrane cargos back to the plasma membrane. An alternative explanation worth being considered is that TORC1 hyperactivation results in the active inhibition of transporter recycling. Indeed, activated TORC1 is well known to promote the ubiquitylation of transporters, while de-ubiquitylation has been reported to favor the recycling of transporters back to the plasma membrane [18,19,36]. Thus, hyperactivation of TORC1 could potentially result in highly efficient ubiquitylation of both cargo proteins and adaptors, thus overriding de-ubiquitylation and recycling. Another aspect worth being considered is that TORC1 components have recently been reported to localize to endosomes and to have specialized signaling functions [78], while the Rag/Gtr GTPases involved in TORC1 activation have additionally been reported to play an important role in the recycling of specific cargos from endosomes to the cell surface [79]. It is thus tempting to speculate that this functionally and spatially distinct pool of Rag/Gtr-TORC1, when hyperactivated upon CHX treatment, might locally control the ubiquitylation and/or recycling of transporters.

## 4. Materials and Methods

### 4.1. Yeast Strains, Plasmids and Growth Conditions

Strains used in this study (Supplementary Table S1) derive from Sigma1278b. The *npi1-1* strain has a Ty element inserted in the promoter of *RSP5*, resulting in many-fold reduced expression [6]. Cells were grown at 29 °C on a minimal buffered medium, pH 6.1 [80], with galactose (Gal; 2–3%) or glucose (Glu; 3%) as carbon source. Nitrogen sources added in the growth media were ammonium, in the form of (NH4)_2_SO_4_ (10 mM), or L-Proline (10 mM). The final concentrations of substances added were arginine (5 mM), (NH4)_2_SO_4_ (10 mM), rapamycin (200 ng/mL), and cycloheximide (50 μg/mL). The *CAN1-GFP* genes were expressed from centromeric plasmids under the control of the *GAL* promoter. Cells were grown on galactose medium, and glucose was added for 0.5 or 1.0 h, in order to allow all Can1-GFP molecules to reach the plasma membrane [17], before cell transfer to conditions inducing transporter ubiquitylation/endocytosis. The plasmids used in this study are listed in Supplementary Table S2. New plasmids were constructed by in vivo homologous recombination in yeast, as previously described [29,81], cloned by rescuing in *Escherichia coli* and verified by sequencing. Nucleotide sequences are available upon request.

### 4.2. Epifluorescence Microscopy

The subcellular location of Can1-GFP in Athens was visualized with an Axioplan 2 (Carl Zeiss, Inc.) epifluorescence microscope equipped with Plan-Apochromat x100 1.40 NA oil immersion objective lens, and appropriate fluorescence light filter sets. Images were captured with a Retiga3 (QImaging, Surrey, BC, Canada) digital camera and Occular 2.01 acquisition software (Teledyne Photometrics, Tucson, AZ, USA). Epifluoresence microscopy acquisition in Gosselies was performed with an Eclipse 80i (Nikon) epifluorescence microscope equipped with a 100× differential interference contrast, numerical aperture (NA) 1.40 Plan-Apochromat objective (Nikon, Tokio, Japan) and appropriate fluorescence light filter sets. Images were captured with a DS-Qi1Mc-U3 (Nikon) digital camera and NIS-Elements 4.3 acquisition software (Nikon). Images were processed with FIJI software (National Institutes of Health, Bethesda, MD, USA) [82] and annotated with Photoshop (Adobe Systems, San Jose, CA, USA). Cells from early logarithmic phase liquid cultures were laid on a thin layer of 1% agar supplemented with appropriate nutrients and observed at room temperature. In each figure, only a few representative cells, observed in at least two independent biological replicate experiments, are shown. In key experiments, fluorescence intensity was quantified.

### 4.3. Fluorescence Quantification and Statistical Analysis

The fluorescence intensity of Can1-GFP was quantified using FIJI software [82], as before [29]. Briefly, two homocentric ellipses outlining the whole cell or everything but the plasma membrane (PM) were manually drawn. The ratio of PM-to-internal-mean-pixel intensity for each cell population is presented in box-and-whisker plots. The values obtained underestimate the actual ratios, because the internal fluorescence also includes background cell fluorescence due to epifluorescence microscopy. Prism software, one-way analysis of variance (ANOVA) with the nonparametric Kruskal–Wallis test, and Dunn’s multiple-comparison post hoc analyses were used to assess the significance of PM-to-internal-fluorescence ratio value differences.

### 4.4. Protein Extracts and Western Blotting

Total cell protein extracts were prepared and analyzed by SDS–PAGE as previously described [6]. Proteins were transferred to either a nitrocellulose membrane (Protran; Perkin Elmer, Waltham, MA, USA) or PVDF (Immobilon, Macherey Nagel, Düren, NW, Germany) and probed with a mouse monoclonal anti-GFP (Roche, Basel, Switzerland), anti-hemagglutinin (anti-HA) (Roche, Basel, Switzerland), anti-yeast 3-phosphoglycerate kinase (anti-PGK; Invitrogen), or anti-actin (sc47778, Santa Cruz Biotechnology, Dallas, TX, USA). Primary antibodies were detected by enhanced chemiluminescence (Roche, Basel, Switzerland, or Millipore, Burlington, MA, USA) after treatment with horseradish-peroxidase-conjugated anti-mouse immunoglobulin (Ig) G secondary antibody (Sigma-Aldrich, St. Louis, MO, USA, or Cell Signalling, Danvers, MA, USA). Signals were detected with CL-Xposure film (Thermo-Fischer Scientific, Waltham, MA, USA) or ImageQuant LAS 4000 mini (FujiFilm, Tokio, Japan). Films were scanned and annotated with FIJI.

### 4.5. Quantification and Statistical Analysis of Western Blots 

Relative semi-quantitative amounts of total Can1-GFP and Ub-Can1-GFP were estimated from 1–8 biological replicates of non-saturated exposures using the gel analyzer tool of FIJI [82]. For this purpose, each respective band was selected by using rectangular ROI selection and “Gels” analyzer, followed by quantification of the peak area of obtained histograms. Data were acquired as area values. In each graph, the ratios of Ub-Can1-GFP /total Can1-GFP are plotted in scatter dot plots. Ratios are zero in cases where the Ub-Can1-GFP signal was not detected. Unpaired two-tailed Student’s T-tests with parametric analyses (Prism, Graphpad, San Diego, CA, USA) were used to assess the significance of Ub-Can1-GFP/total Can1-GFP ratio value differences.

## Supplementary Materials

The Supplementary Materials are available online at https://www.mdpi.com/article/10.3390/ijms221910208/s1.

## References

[1] Kaback H.R., Smirnova I., Kasho V., Nie Y., Zhou Y. (2011). The Alternating Access Transport Mechanism in LacY. J. Membr. Biol..

[2] Müller-Hill B. (2011). The Lac Operon: A Short History of a Genetic Paradigm.

[3] Haguenauer-Tsapis R., André B. (2004). Membrane trafficking of yeast transporters: Mechanisms and physiological control of downregulation. Molecular Mechanisms Controlling Transmembrane Transport.

[4] Kahlhofer J., Leon S., Teis D., Schmidt O. (2021). The α-arrestin family of ubiquitin ligase adaptors links metabolism with selective endocytosis. Biol. Cell.

[5] Lauwers E., Erpapazoglou Z., Haguenauer-Tsapis R., André B. (2010). The ubiquitin code of yeast permease trafficking. Trends. Cell. Biol..

[6] Hein C., Springael J.-Y., Volland C., Haguenauer-Tsapis R., André B. (1995). NPI1, an essential yeast gene involved in induced degradation of Gap1 and Fur4 permeases, encodes the Rsp5 ubiquitin—Protein ligase. Mol. Microbiol..

[7] Kee Y., Muñoz W., Lyon N., Huibregtse J.M. (2006). The deubiquitinating enzyme Ubp2 modulates Rsp5-dependent Lys 63-linked polyubiquitin conjugates in Saccharomyces cerevisiae. J. Biol. Chem..

[8] Lauwers E., Jacob C., Andre B. (2009). K63-linked ubiquitin chains as a specific signal for protein sorting into the multivesicular body pathway. J. Cell Biol..

[9] Henne W.M., Buchkovich N.J., Emr S.D. (2011). The ESCRT Pathway. Dev. Cell.

[10] Lin C.H., MacGurn J.A., Chu T., Stefan C.J., Emr S.D. (2008). Arrestin-Related Ubiquitin-Ligase Adaptors Regulate Endocytosis and Protein Turnover at the Cell Surface. Cell.

[11] Barata-Antunes C., Alves R., Talaia G., Casal M., Gerós H., Mans R., Paiva S. (2021). Endocytosis of nutrient transporters in fungi: The ART of connecting signaling and trafficking. Comput. Struct. Biotechnol. J..

[12] O’donnell A.F., Schmidt M.C. (2019). AMPK-mediated regulation of alpha-arrestins and protein trafficking. Int. J. Mol. Sci..

[13] Becuwe M., Herrador A., Haguenauer-Tsapis R., Vincent O., Léon S. (2012). Ubiquitin-Mediated Regulation of Endocytosis by Proteins of the Arrestin Family. Biochem. Res. Int..

[14] Nikko E., Pelham H.R. (2009). Arrestin-mediated endocytosis of yeast plasma membrane transporters. Traffic.

[15] Becuwe M., Vieira N., Lara D., Gomes-Rezende J., Soares-Cunha C., Casal M., Haguenauer-Tsapis R., Vincent O., Paiva S., Léon S. (2012). A molecular switch on an arrestin-like protein relays glucose signaling to transporter endocytosis. J. Cell Biol..

[16] Merhi A., André B. (2012). Internal Amino Acids Promote Gap1 Permease Ubiquitylation via TORC1/Npr1/14-3-3-Dependent Control of the Bul Arrestin-Like Adaptors. Mol. Cell. Biol..

[17] Ghaddar K., Merhi A., Saliba E., Krammer E.-M., Prévost M., André B. (2014). Substrate-Induced Ubiquitylation and Endocytosis of Yeast Amino Acid Permeases. Mol. Cell. Biol..

[18] MacDonald C., Shields S.B., Williams C.A., Winistorfer S., Piper R.C. (2020). A Cycle of Ubiquitination Regulates Adaptor Function of the Nedd4-Family Ubiquitin Ligase Rsp5. Curr. Biol..

[19] Ho H.C., MacGurn J.A., Emr S.D. (2017). Deubiquitinating enzymes Ubp2 and Ubp15 regulate endocytosis by limiting ubiquitination and degradation of ARTs. Mol. Biol. Cell.

[20] MacGurn J.A.A., Hsu P.-C.C., Smolka M.B.B., Emr S.D.D. (2011). TORC1 regulates endocytosis via npr1-mediated phosphoinhibition of a ubiquitin ligase adaptor. Cell.

[21] Martínez-Márquez J.Y., Duncan M.C. (2018). Investigation of Ldb19/Art1 localization and function at the late Golgi. PLoS ONE.

[22] Becuwe M., Léon S. (2014). Integrated control of transporter endocytosis and recycling by the arrestin-related protein Rod1 and the ubiquitin ligase Rsp5. Elife.

[23] O’Donnell A.F., Huang L., Thorner J., Cyert M.S. (2013). A calcineurin-dependent switch controls the trafficking function of α-arrestin Aly1/Art6. J. Biol. Chem..

[24] Toyoda Y., Soejima S., Masuda F., Saitoh S. (2021). TORC2 inhibition of α-arrestin Aly3 mediates cell surface persistence of S. pombe Ght5 glucose transporter in low glucose. J. Cell Sci..

[25] Paiva S., Vieira N., Nondier I., Haguenauer-Tsapis R., Casal M., Urban-Grimal D. (2009). Glucose-induced ubiquitylation and endocytosis of the yeast Jen1 transporter. Role of lysine 63-linked ubiquitin chains. J. Biol. Chem..

[26] Laidlaw K.M.E., Bisinski D.D., Shashkova S., Paine K.M., Veillon M.A., Leake M.C., MacDonald C. (2021). A glucose-starvation response governs endocytic trafficking and eisosomal retention of surface cargoes in budding yeast. J. Cell Sci..

[27] Savocco J., Nootens S., Afokpa W., Bausart M., Chen X., Villers J., Renard H.F., Prévost M., Wattiez R., Morsomme P. (2019). Yeast α-arrestin Art2 is the key regulator of ubiquitylation-dependent endocytosis of plasma membrane vitamin B1 transporters. PLoS Biol..

[28] Karachaliou M., Amillis S., Evangelinos M., Kokotos A.C., Yalelis V., Diallinas G. (2013). The arrestin-like protein ArtA is essential for ubiquitination and endocytosis of the UapA transporter in response to both broad-range and specific signals. Mol. Microbiol..

[29] Gournas C., Saliba E., Krammer E.-M., Barthelemy C., Prévost M., André B. (2017). Transition of yeast Can1 transporter to the inward-facing state unveils an α-arrestin target sequence promoting its ubiquitylation and endocytosis. Mol. Biol. Cell.

[30] Guiney E.L., Klecker T., Emr S.D. (2016). Identification of the endocytic sorting signal recognized by the Art1-Rsp5 ubiquitin ligase complex. Mol. Biol. Cell..

[31] Fujita S., Sato D., Kasai H., Ohashi M., Tsukue S., Takekoshi Y., Gomi K., Shintani T. (2018). The C-terminal region of the yeast monocarboxylate transporter Jen1 acts as a glucose signal–responding degron recognized by the α-arrestin Rod1. J. Biol. Chem..

[32] Ivashov V., Zimmer J., Schwabl S., Kahlhofer J., Weys S., Gstir R., Jakschitz T., Kremser L., Bonn G.K., Lindner H. (2020). Complementary α-arrestin-ubiquitin ligase complexes control nutrient transporter endocytosis in response to amino acids. Elife.

[33] Lee S., Ho H.-C., Tumolo J.M., Hsu P.-C., MacGurn J.A. (2019). Methionine triggers Ppz-mediated dephosphorylation of Art1 to promote cargo-specific endocytosis. J. Cell. Biol..

[34] Baile M.G., Guiney E.L., Sanford E.J., Macgurn J.A., Smolka M.B., Emr S.D. (2019). Activity of a ubiquitin ligase adaptor is regulated by disordered insertions in its arrestin domain. Mol. Biol. Cell.

[35] Schmitz C., Kinner A., Kölling R. (2005). The deubiquitinating enzyme Ubp1 affects sorting of the ATP-binding cassette-transporter ste6 in the endocytic pathway. Mol. Biol. Cell.

[36] Suresh H.G., Pascoe N., Andrews B. (2020). The structure and function of deubiquitinases: Lessons from budding yeast. Open Biol..

[37] Boase N.A., Kelly J.M. (2004). A role for creD, a carbon catabolite repression gene from Aspergillus nidulans, in ubiquitination. Mol. Microbiol..

[38] Lam M.H.Y., Urban-Grimal D., Bugnicourt A., Greenblatt J.F., Haguenauer-Tsapis R., Emili A. (2009). Interaction of the deubiquitinating enzyme Ubp2 and the e3 ligase Rsp5 is required for transporter/receptor sorting in the multivesicular body pathway. PLoS ONE.

[39] Nikko E., Marini A.-M.M., André B. (2003). Permease Recycling and Ubiquitination Status Reveal a Particular Role for Bro1 in the Multivesicular Body Pathway. J. Biol. Chem..

[40] Grossmann G., Malinsky J., Stahlschmidt W., Loibl M., Weig-Meckl I., Frommer W.B., Opekarova M., Tanner W., Opekarová M., Tanner W. (2008). Plasma membrane microdomains regulate turnover of transport proteins in yeast. J. Cell Biol..

[41] Zhao Y., MacGurn J.A., Liu M., Emr S. (2013). The ART-Rsp5 ubiquitin ligase network comprises a plasma membrane quality control system that protects yeast cells from proteotoxic stress. Elife.

[42] Crapeau M., Merhi A., André B. (2014). Stress conditions promote yeast Gap1 permease ubiquitylation and down-regulation via the arrestin-like bul and aly proteins. J. Biol. Chem..

[43] Buelto D., Hung C.W., Aoh Q.L., Lahiri S., Duncan M.C. (2020). Plasma membrane to vacuole traffic induced by glucose starvation requires Gga2-dependent sorting at the trans-Golgi network. Biol. Cell.

[44] Mikros E., Diallinas G. (2019). Tales of tails in transporters. Open Biol..

[45] Keener J.M., Babst M. (2013). Quality Control and Substrate-Dependent Downregulation of the Nutrient Transporter Fur4. Traffic.

[46] Hovsepian J., Albanèse V., Becuwe M., Ivashov V., Teis D., Léon S. (2018). The yeast arrestin-related protein Bul1 is a novel actor of glucose-induced endocytosis. Mol. Biol. Cell.

[47] Talaia G., Gournas C., Saliba E., Barata-Antunes C., Casal M., André B., Diallinas G., Paiva S. (2017). The α-Arrestin Bul1p Mediates Lactate Transporter Endocytosis in Response to Alkalinization and Distinct Physiological Signals. J. Mol. Biol..

[48] Binda M., Péli-Gulli M.-P., Bonfils G., Panchaud N., Urban J., Sturgill T.W., Loewith R., De Virgilio C. (2009). The Vam6 GEF controls TORC1 by activating the EGO complex. Mol. Cell.

[49] Santos D.A., Shi L., Tu B.P., Weissman J.S. (2019). Cycloheximide can distort measurements of mRNA levels and translation efficiency. Nucleic Acids Res..

[50] Grenson M., Crabeel M., Wiame J.M., Béchet J. (1968). Inhibition of protein synthesis and simulation of permease turnover in yeast. Biochem. Biophys. Res. Commun..

[51] Wiame J.M., Grenson M., Arst H.N. (1985). Nitrogen Catabolite Repression in Yeasts and Filamentous Fungi. Adv. Microb. Physiol..

[52] Godard P., Urrestarazu A., Vissers S., Kontos K., Bontempi G., van Helden J., André B. (2007). Effect of 21 Different Nitrogen Sources on Global Gene Expression in the Yeast *Saccharomyces cerevisiae*. Mol. Cell. Biol..

[53] Magasanik B., Kaiser C.A. (2002). Nitrogen regulation in Saccharomyces cerevisiae. Gene.

[54] Roberg K.J., Rowley N., Kaiser C.A. (1997). Physiological Regulation of Membrane Protein Sorting Late in the Secretory Pathway of Saccharomyces cerevisiae. J. Cell Biol..

[55] Day K.J., Casler J.C., Glick B.S. (2018). Budding Yeast Has a Minimal Endomembrane System. Dev. Cell.

[56] Schmidt A., Beck T., Koller A., Kunz J., Hall M.N. (1998). The TOR nutrient signalling pathway phosphorylates NPR1 and inhibits turnover of the tryptophan permease. EMBO J..

[57] Saliba E., Evangelinos M., Gournas C., Corrillon F., Georis I., André B. (2018). The yeast H+-ATPase Pma1 promotes Rag/Gtr-dependent TORC1 activation in response to H+-coupled nutrient uptake. Elife.

[58] González A., Hall M.N. (2017). Nutrient sensing and TOR signaling in yeast and mammals. EMBO J..

[59] Luo Z., Gallwitz D. (2003). Biochemical and genetic evidence for the involvement of yeast Ypt6-GTPase in protein retrieval to different Golgi compartments. J. Biol. Chem..

[60] Gournas C., Gkionis S., Carquin M., Twyffels L., Tyteca D., André B. (2018). Conformation-dependent partitioning of yeast nutrient transporters into starvation-protective membrane domains. Proc. Natl. Acad. Sci. USA.

[61] Grenson M., Mousset M., Wiame J.M., Bechet J. (1966). Multiplicity of the amino acid permeases in Saccharomyces cerevisiae: I. Evidence for a specific arginine-transporting system. Biochim. Biophys. Acta.

[62] Malínská K., Malínský J., Opekarová M., Tanner W. (2003). Visualization of Protein Compartmentation within the Plasma Membrane of Living Yeast Cells. Mol. Biol. Cell.

[63] Busto J.V., Elting A., Haase D., Spira F., Kuhlman J., Schäfer-Herte M., Wedlich-Söldner R., Schäfer-Herte M., Wedlich-Söldner R., Schäfer-Herte M. (2018). Lateral plasma membrane compartmentalization links protein function and turnover. EMBO J..

[64] Moharir A., Gay L., Appadurai D., Keener J., Babst M. (2018). Eisosomes are metabolically regulated storage compartments for APC-type nutrient transporters. Mol. Biol. Cell.

[65] Babst M. (2019). Eisosomes at the Intersection of TORC1 and TORC2 Regulation. Traffic.

[66] Bianchi F., Syga Ł., Moiset G., Spakman D., Schavemaker P.E., Punter C.M., Seinen A.-B., van Oijen A.M., Robinson A., Poolman B. (2018). Steric exclusion and protein conformation determine the localization of plasma membrane transporters. Nat. Commun..

[67] Busto J.V., Wedlich-Söldner R. (2019). Integration Through Separation—The Role of Lateral Membrane Segregation in Nutrient Uptake. Front. Cell Dev. Biol..

[68] Athanasopoulos A., André B., Sophianopoulou V., Gournas C. (2019). Fungal plasma membrane domains. FEMS Microbiol. Rev..

[69] Nishimura A., Tanahashi R., Takagi H. (2020). The yeast α-arrestin Art3 is a key regulator for arginine-induced endocytosis of the high-affinity proline transporter Put4. Biochem. Biophys. Res. Commun..

[70] Vandenbol M., Jauniaux J.C., Grenson M. (1989). Nucleotide sequence of the Saccharomyces cerevisiae PUT4 proline-permease-encoding gene: Similarities between CAN1, HIP1 and PUT4 permeases. Gene.

[71] Jauniaux J.-C., Grenson M. (1990). GAP1, the general amino acid permease gene of Saccharomyces cerevisiae Nucleotide sequence, protein similarity with the other bakers yeast amino acid permeases, and nitrogen catabolite repression. Eur. J. Biochem..

[72] Brach T., Specht T., Kaksonen M. (2011). Reassessment of the role of plasma membrane domains in the regulation of vesicular traffic in yeast. J. Cell Sci..

[73] Grenson M., Mousset M., Wiame J.M., Bechet J. (1966). Multiplicity of the amino acid permeases in Saccharomyces cerevisiae: II. Evidence for a specific lysine-transporting system. Biochim. Biophys. Acta.

[74] Villers J., Savocco J., Szopinska A., Degand H., Nootens S., Morsomme P. (2017). Study of the Plasma Membrane Proteome Dynamics Reveals Novel Targets of the Nitrogen Regulation in Yeast. Mol. Cell. Proteom..

[75] Malecki M., Kamrad S., Ralser M., Bähler J. (2020). Mitochondrial respiration is required to provide amino acids during fermentative proliferation of fission yeast. EMBO Rep..

[76] Olin-Sandoval V., Yu J.S.L., Miller-Fleming L., Alam M.T., Kamrad S., Correia-Melo C., Haas R., Segal J., Peña Navarro D.A., Herrera-Dominguez L. (2019). Lysine harvesting is an antioxidant strategy and triggers underground polyamine metabolism. Nature.

[77] Ruiz S.J., van ’t Klooster J.S., Bianchi F., Poolman B. (2021). Growth inhibition by amino acids in saccharomyces cerevisiae. Microorganisms.

[78] Hatakeyama R., Péli-Gulli M.P., Hu Z., Jaquenoud M., Garcia Osuna G.M., Sardu A., Dengjel J., De Virgilio C. (2019). Spatially Distinct Pools of TORC1 Balance Protein Homeostasis. Mol. Cell.

[79] MacDonald C., Piper R.C. (2017). Genetic dissection of early endosomal recycling highlights a TORC1-independent role for Rag GTPases. J. Cell Biol..

[80] Jacobs P., Jauniaux J.-C., Grenson M. (1980). A cis-dominant regulatory mutation linked to the argB-argC gene cluster in Saccharomyces cerevisiae. J. Mol. Biol..

[81] Merhi A., Gérard N., Lauwers E., Prévost M., André B. (2011). Systematic mutational analysis of the intracellular regions of yeast gap1 permease. PLoS ONE.

[82] Schindelin J., Arganda-Carreras I., Frise E., Kaynig V., Longair M., Pietzsch T., Preibisch S., Rueden C., Saalfeld S., Schmid B. (2012). Fiji: An open-source platform for biological-image analysis. Nat. Methods.

