# Supplementary figures and images for: The Bul1/2 Alpha-Arrestins Promote Ubiquitylation and Endocytosis of the Can1 Permease upon Cycloheximide-Induced TORC1-Hyperactivation
Source: Int J Mol Sci. 2021 Sep 22;22(19):10208. doi: 10.3390/ijms221910208 (PMC8508209; doi:10.3390/ijms221910208)

Figure 2B

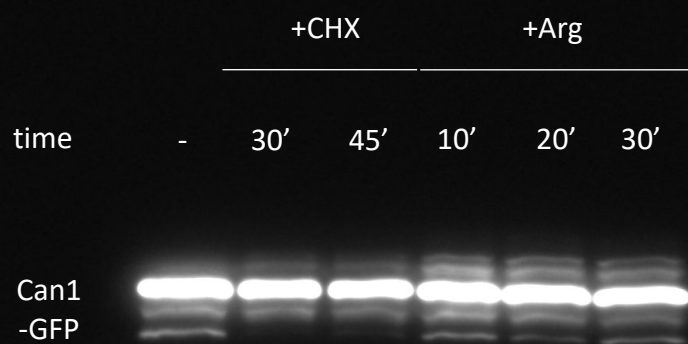

Figure 2B

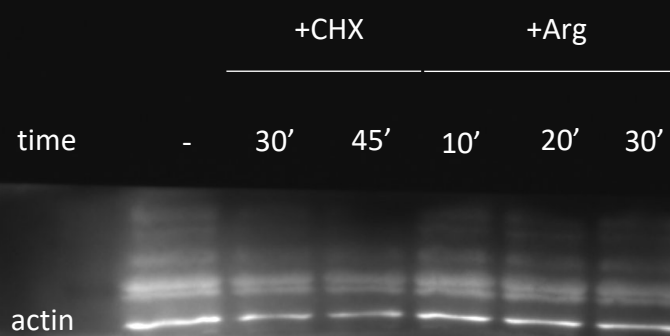

Figure 2C

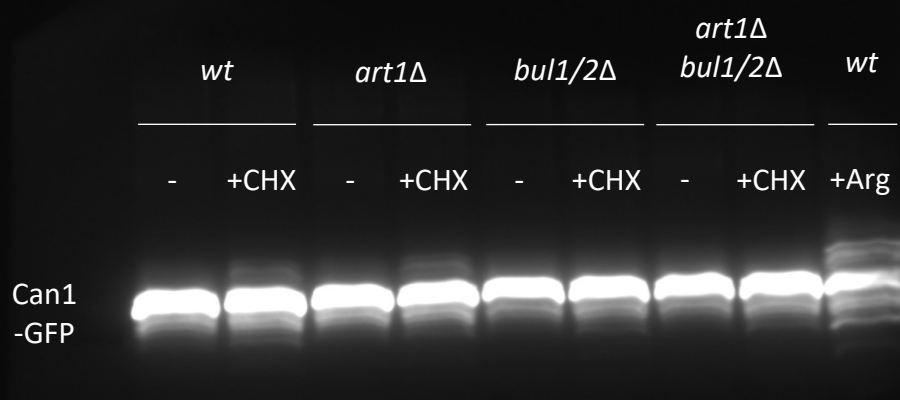

Figure 2C

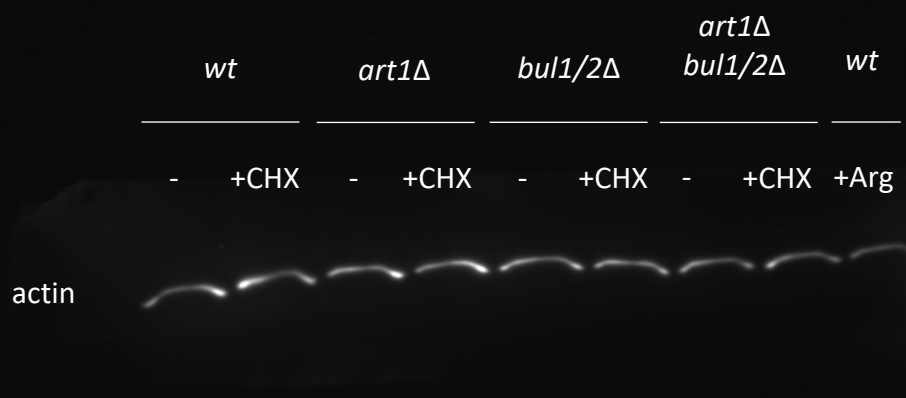

Figure 3D

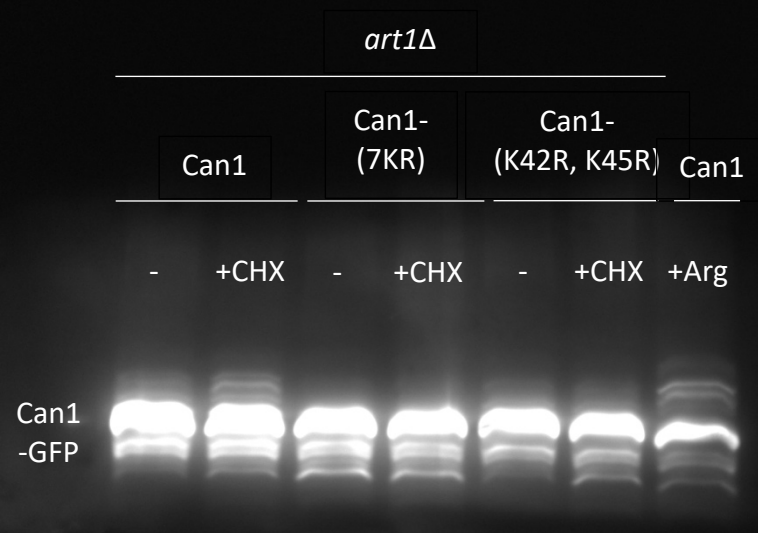

Figure 3D

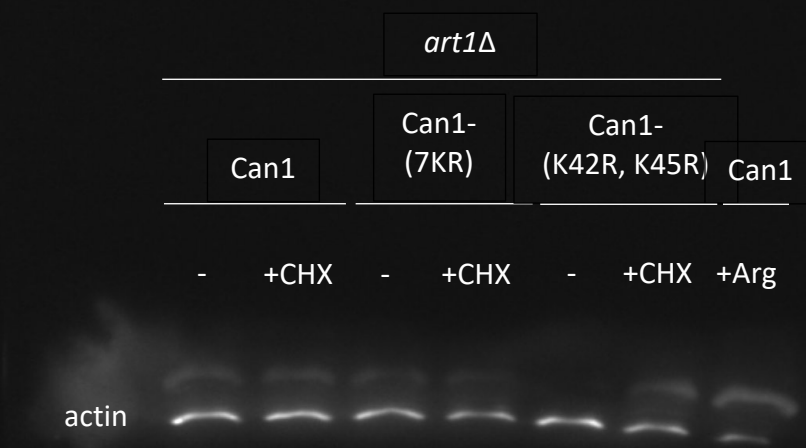

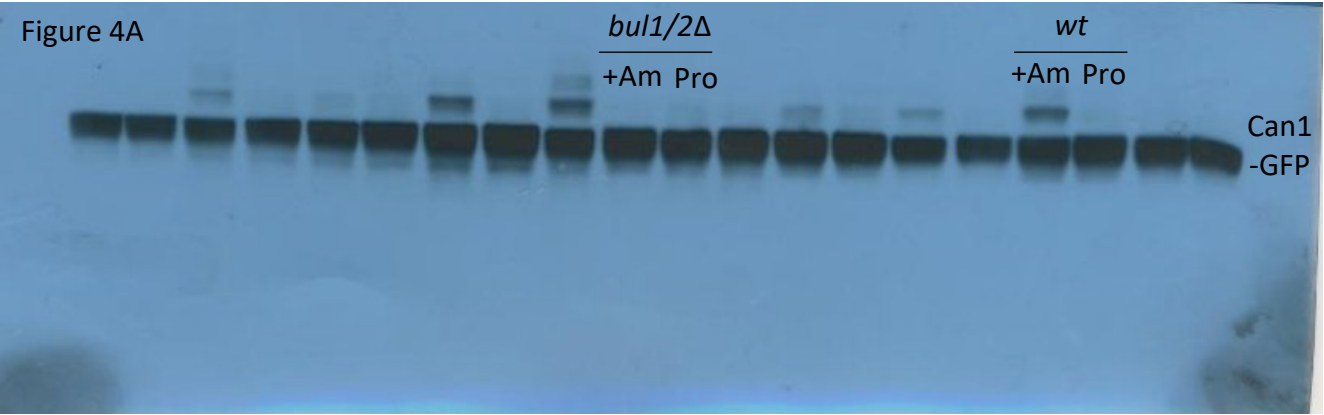

Figure 4B

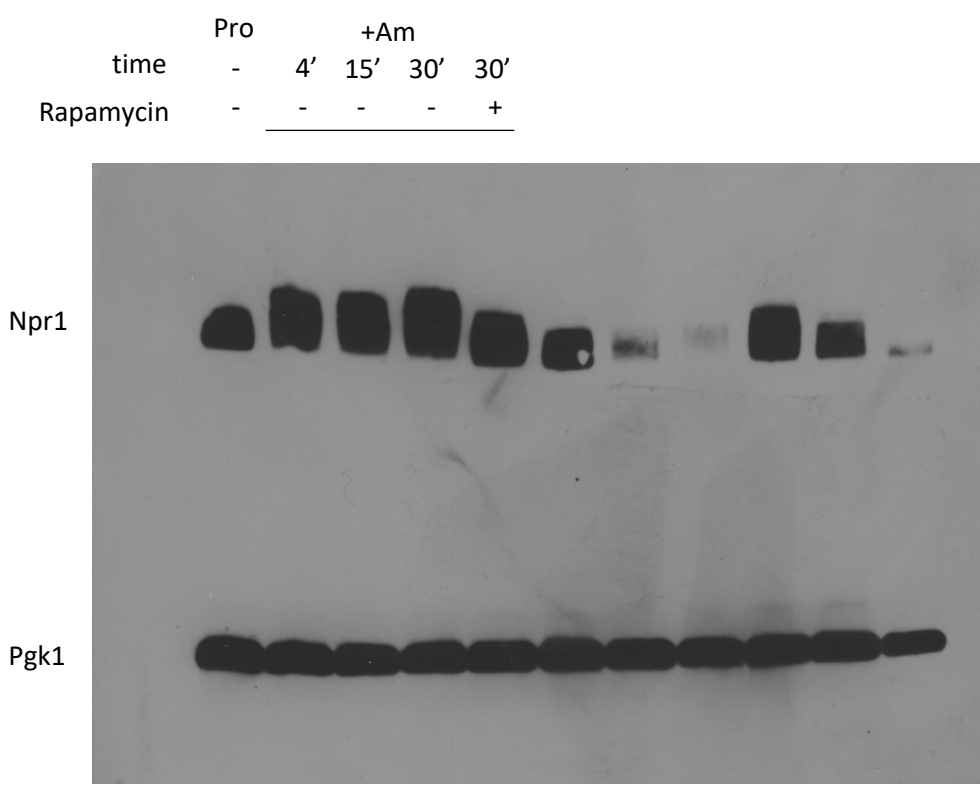

Figure 4C

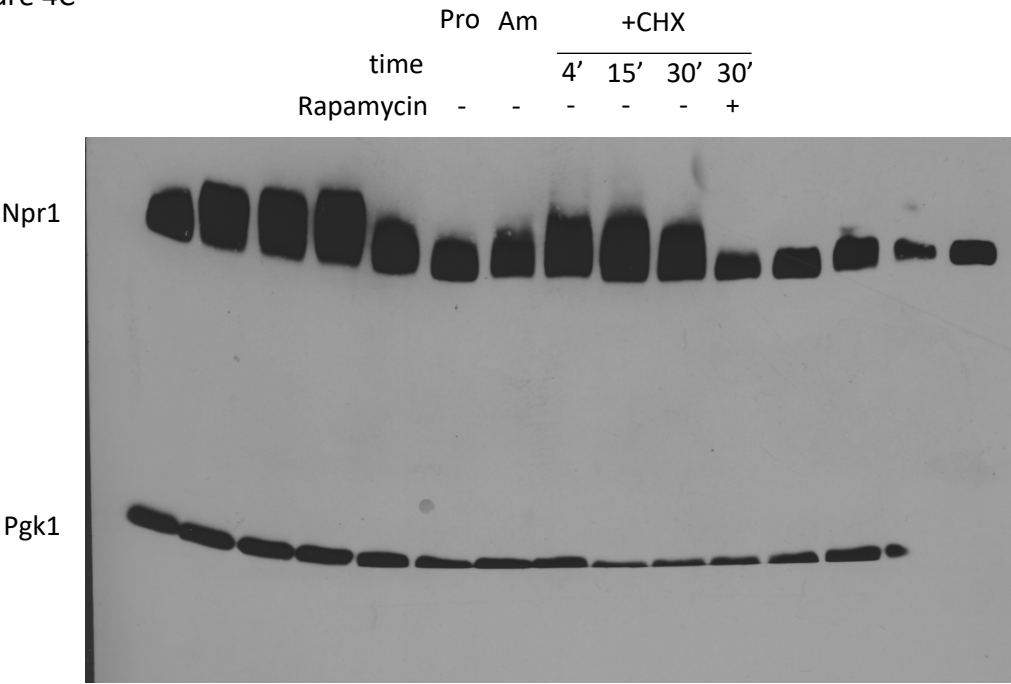

Supplement: Supplementary file 1 [file ijms-22-10208-s001.zip › ijms-1327241-supplementary.pdf]
